# Monitoring ADAMTS-13 conformation in immune-mediated thrombotic thrombocytopenic purpura: toward personalized management

**DOI:** 10.1016/j.rpth.2025.103288

**Published:** 2025-12-09

**Authors:** Bérangère S. Joly, Elien Roose, Charlotte Dekimpe, Karen Vanhoorelbeke, Agnès Veyradier, Paul Coppo

**Affiliations:** 1 Service d'Hématologie biologique, Hôpital Lariboisière, AP-HP.Nord, Université Paris Cité, Paris, France; 2 Université Paris Cité, Sorbonne Université, INSERM UMRS1138, Centre de Recherche des Cordeliers, Paris, France; 3 Laboratory for Thrombosis Research, KU Leuven Campus Kulak Kortrijk, Kortrijk, Belgium; 4 Service d’Hématologie, Centre de référence des microangiopathies thrombotiques (CNR-MAT), Hôpital Saint-Antoine, AP-HP.Sorbonne Université (AP-HP.6), Paris, France

**Keywords:** ADAMTS-13 protein, protein conformations, thrombotic microangiopathy, thrombotic thrombocytopenic purpura

## Abstract

**Background:**

Immune-mediated thrombotic thrombocytopenic purpura (iTTP) is a life-threatening thrombotic microangiopathy caused by an autoimmune-driven deficiency of ADAMTS-13. Despite remission, relapses remain a major concern for patients and are currently predicted by monitoring ADAMTS-13 activity.

**Objectives:**

This study evaluated the association between ADAMTS-13 conformation and relapse risk in patients with iTTP during follow-up.

**Methods:**

We conducted a retrospective monocentric study involving patients with iTTP with ADAMTS-13 monitoring from 2008 to 2020. ADAMTS-13 antigen and conformation were assessed in plasma samples using our 3H9-ELISA and 1C4-ELISA, respectively.

**Results:**

Fifteen patients with iTTP were monitored for a median of 7 years (IQR, 6-11) with a total of 479 plasma samples. Based on annual relapse rate (RR; median, 0.5), they were categorized as low (group 1; RR, <0.50, *n* = 8) or high relapsers (group 2; RR, ≥0.50, *n* = 7). ADAMTS-13 activity normalized between iTTP relapses in all patients. However, the time from normalization with an open conformation to relapse was shorter in group 2 (5 vs 21 months; *P* < .001). Median annual ADAMTS-13 activity differ significantly between groups (54.1% vs 50.0%; *P* = .1893). A trend suggested greater time spent in an open conformation in group 2 (0.6 vs 0.2; *P* = .1427). Rituximab was effective in group 1, while group 2 patients often required alternative therapies.

**Conclusion:**

Persistent open ADAMTS-13 conformation in remission samples was observed more frequently in patients with higher relapse risk and could potentially serve as a biomarker for detecting low levels of circulating autoantibodies. This potential biomarker requires prospective validation before it can be used to guide individualized iTTP management.

## 1 Introduction

Conformational changes in self-proteins able to disrupt self-tolerance are evidenced in several autoimmune disease models, including antiphospholipid syndrome [1], heparin-induced thrombocytopenia [2], and thrombotic thrombocytopenic purpura (TTP) [3]. ADAMTS-13 (a disintegrin and metalloprotease with thrombospondin type 1 motifs, member 13), a metalloprotease involved in the pathophysiology of TTP, is a multidomain glycoprotein (M, D, T, C, S, T2T8, and CUB1-CUB2 domains) circulating in a latent, closed conformation, maintained by self-interaction between its spacer (S) and CUB domains. Upon physiological interaction with its substrate, von Willebrand factor (VWF), this autoinhibition between the spacer and CUB domains is disrupted, leading to ADAMTS-13 adopting a transient open conformation. The presence of 3 linker regions between the distal T domains contributes to the flexibility of ADAMTS-13 [4].

Immune-mediated TTP (iTTP) results from an autoimmune-driven severe deficiency of ADAMTS-13 (plasma activity, <10 IU/dL) [5]. The immune response in iTTP is polyclonal, but the majority of patients have autoantibodies targeting the CS region, identifying a cryptic epitope within the S domain [[6], [7], [8]]. This cryptic epitope is protected by CUB domains when ADAMTS-13 is in its closed conformation, while it is exposed when ADAMTS-13 adopts an open conformation [9]. Recently, the study of ADAMTS-13 conformation has emerged as an innovative biological parameter that has gained significance in understanding the pathophysiology of iTTP [3,10,11]. Interestingly, an open conformation of ADAMTS-13 has been demonstrated as a hallmark of iTTP at the acute phase, whereas a closed conformation is observed during remission after patients have recovered a normal ADAMTS-13 activity. In that regard, anti–ADAMTS-13 antibodies were reported to induce an open ADAMTS-13 conformation [3,12]; however, whether anti–ADAMTS-13 antibodies alter ADAMTS-13 conformation and activity simultaneously or through a multistep process remains unclear. Hence, it could be hypothesized that anti–ADAMTS-13 antibodies induce first the opening of ADAMTS-13, exposing the immunogenic cryptic site, followed by the production of additional anti–ADAMTS-13 antibodies, further altering ADAMTS-13 conformation and activity.

The standard treatment for acute iTTP episodes requires a combination of therapeutic plasma exchange (TPE), immunomodulation (corticosteroids and an anti-CD20 monoclonal antibody, rituximab), and more recently, the anti-VWF nanobody caplacizumab [13,14]. However, iTTP survivors are exposed to unpredictable relapses of potentially severe outcome [15,16] as well as to treatment-related complications [5,14,17,18]. Currently, ADAMTS-13 activity is the only biomarker used to predict relapses in patients with iTTP monitored during remission: in that regard, an ADAMTS-13 activity of <20 IU/dL (defining ADAMTS-13 relapse) [13,17] usually results in pre-emptive immunomodulation to prevent a full-blown clinical relapse [19,20]. In France, patients with iTTP are closely monitored for ADAMTS-13 activity every 3 to 6 months, during the years following the acute phase [20]. The systematic use of pre-emptive rituximab in patients with iTTP with ADAMTS-13 relapse could substantially decrease the incidence of clinical relapse [[20], [21], [22]]. Alternative anti-CD20 treatments, obinutuzumab and ofatumumab, can be used in patients unresponsive or intolerant to rituximab [[23], [24], [25]].

In patients with iTTP, ADAMTS-13 autoantibodies could be an earlier marker than ADAMTS-13 activity for the risk of relapse because, *in vivo*, ADAMTS-13 antibodies are the causal factor in the drop of ADAMTS-13 activity. However, ADAMTS-13 antibodies are not used as a marker of iTTP relapse because the routine techniques for ADAMTS-13 antibodies titration detect only free, unbound anti–ADAMTS-13 IgGs but not those complexed with ADAMTS-13 [26,27]. In contrast, ADAMTS-13 autoantibodies complexed to ADAMTS-13 are likely to be associated with an open conformation of ADAMTS-13, even if they are not yet in sufficient amount to induce a severe functional deficiency of ADAMTS-13. In that regard, ADAMTS-13 conformation has been found open in patients with subclinical iTTP [12]. Thus, by providing indirect evidence of the earliest presence of ADAMTS-13 autoantibodies in patients with iTTP in remission, an open ADAMTS-13 conformation could represent an earlier predictive marker than ADAMTS-13 activity, for the risk of both ADAMTS-13 relapse and iTTP clinical relapse [12,28]. This view has been recently supported in 2 studies where an open ADAMTS-13 conformation occurred before severe ADAMTS-13 deficiency [29]; moreover, the open ADAMTS-13 conformation predicted earlier clinical relapse than ADAMTS-13 activity, especially during peak activity [30]. Based on the aforementioned statements, the aim of the present study was to evaluate the association between changes in ADAMTS-13 conformation over time and relapse in patients with iTTP using extended follow-up data from a national cohort.

## 2 Methods

### 2.1 Patients

Since 2000, all patients with a presumptive diagnosis of thrombotic microangiopathy (TMA) have been prospectively enrolled in the registry of the French Reference Center for TMA. Adult patients previously diagnosed for iTTP (ADAMTS-13 activity, <10 IU/dL and positive anti–ADAMTS-13 IgG) undergoing long-term monitoring of ADAMTS-13 activity (≥12 months) with available plasma samples were included in this retrospective, monocentric study at Saint-Antoine hospital (AP-HP.Sorbonne Université, Paris, France). For a more consistent management and follow-up of patients with iTTP in the era of front-line and pre-emptive rituximab, monitoring was studied from January 2008 to December 2020. Children and pregnant women were excluded from this study. Written informed consent was obtained from all patients in accordance with the Declaration of Helsinki. This study was approved by the Ethics Committee of Hospital Pitié-Salpêtrière (Paris, France) and is registered at www.clinicaltrials.gov under the number NCT00426686 and at the Health Authority and the French Ministry of Health under the number P051064/PHRC AOM05012. Citrated plasma samples were available from the national biobank of TMA (AC-2023-6021, Lariboisière hospital, AP-HP.Nord, Paris, France).

### 2.2 Response definitions

At the acute phase, clinical response to treatment was defined by sustained platelet count of ≥150 × 10^9^/L and lactate dehydrogenase (LDH) of <1.5 times the upper limit of normal. An exacerbation occurs after a clinical response, if platelet count decreases to <150 × 10^9^/L and LDH increase within 30 days of cessation of TPE. The clinical remission is defined by a platelet count that remains ≥150 × 10^9^/L and LDH of <1.5 times upper limit of normal for ≥30 days after cessation of TPE [18]. Clinical relapse was defined by a platelet count decreasing to <150 × 10^9^/L together with an ADAMTS-13 activity of <10 IU/dL, after a clinical remission. ADAMTS-13 relapse was defined by a decreased ADAMTS-13 activity of <20 IU/dL during clinical remission, with a normal platelet count [18].

### 2.3 ADAMTS-13 phenotypic assays

ADAMTS-13 activity and anti–ADAMTS-13 IgG titration were performed as part of the clinical care, using our in-house FRETS-VWF73 assay (normal range, 50-150 IU/dL) and the TECHNOZYM ADAMTS-13 INH ELISA (Technoclone; positivity threshold, >15 U/mL), respectively. ADAMTS-13 antigen (normal range, 0.930-1.350 μg/mL) was assessed in all plasma samples by our in-house 3H9-ELISA, as previously described [3]. ADAMTS-13 conformation was tested in plasma samples with detectable ADAMTS-13 antigen (concentration, ≥0.030 μg/mL) using our in-house 1C4-ELISA, as previously described [3]. A conformation index (CI) of ≥0.5 is defined as open ADAMTS-13 [3].

### 2.4 Statistical analysis

Quantitative parameters are reported as median (IQR); qualitative parameters are reported as numbers and proportions. Statistical comparisons were conducted using the Mann–Whitney U-test or chi-squared test, with significance set at *P* < .05. Analysis was performed using Prism v.8.4.3 (GraphPad Software).

## 3 Results

### 3.1 Longitudinal follow-up and relapse profile

**Figure 1:**
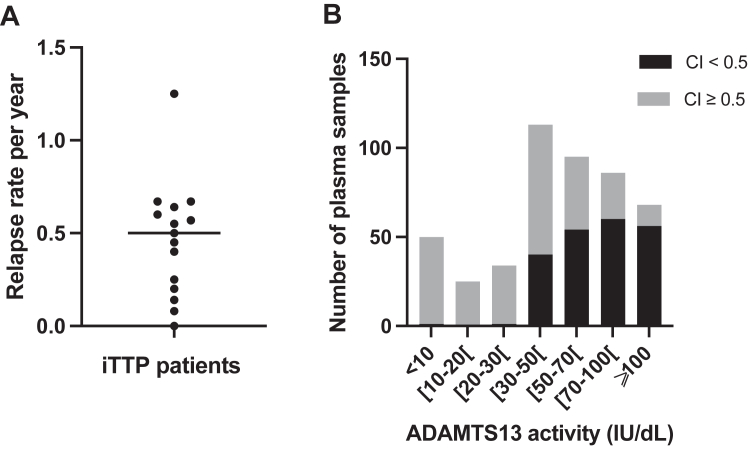
ADAMTS-13 conformation in patients with iTTP included in the kinetic study during their follow-up. (A) The relapse rate per patient with a median at 0.50 relapse per year, defined 2 groups of patients with iTTP (group 1: low relapsers, annual relapse rate < 0.50; group 2: high relapsers, annual relapse rate ≥ 0.50). (B) ADAMTS-13 conformation was measured in 471 plasma samples, with an ADAMTS-13 activity ranging from <10 to 150 IU/dL. ADAMTS-13 conformation was open in 259 samples (55.0%; 107 samples with an activity of <30 IU/dL and 152 samples with an activity of >30 IU/dL). CI, conformation index; iTTP, immune-mediated thrombotic thrombocytopenic purpura.

**Figure 2:**
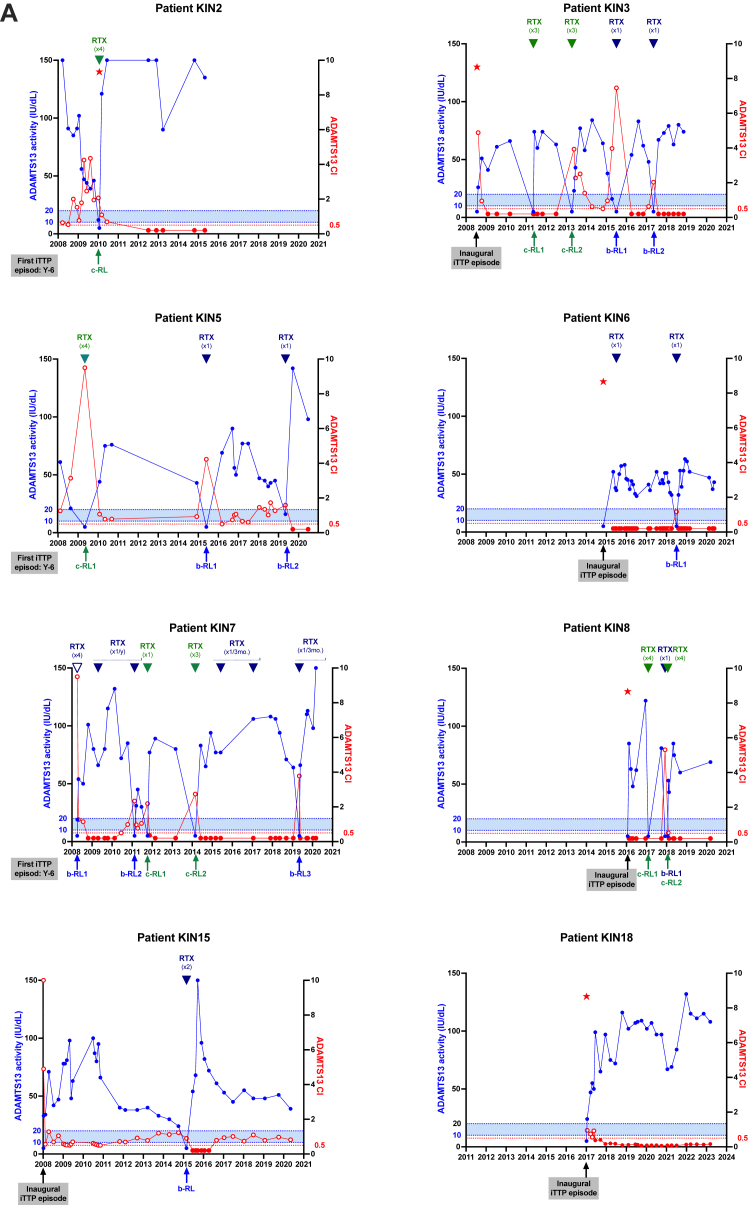

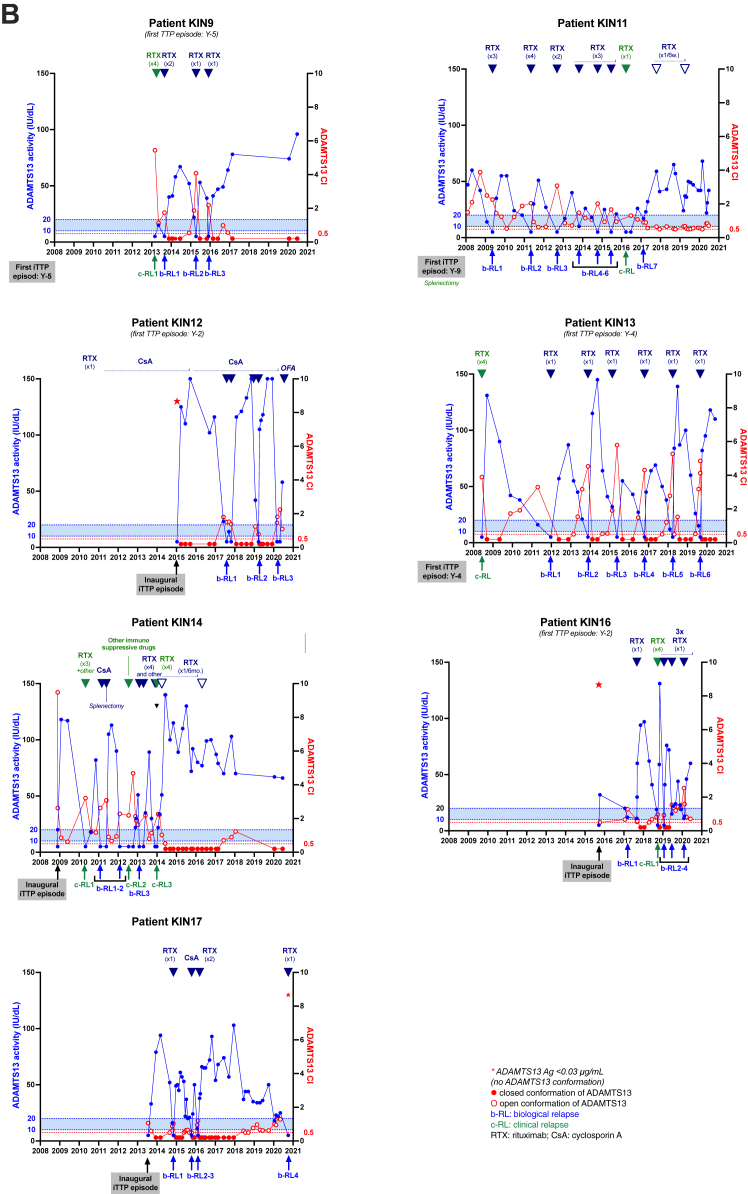
Kinetic study of ADAMTS-13 activity and conformation during long-term monitoring of 15 patients with iTTP. Phenotypic assays (activity, antigen, and conformation) were assessed in plasma samples of 15 patients with iTTP, 8 patients of group 1 (A) and 7 patients of group 2 (B). ADAMTS-13 activity, represented in blue, was measured using the reference method FRETS-VWF73. The dotted blue line indicates the biological threshold for TTP diagnosis (activity, <10 IU/dL), and relapse (activity, 10-20 IU/dL) ADAMTS-13 conformation, represented in red, was measured using the 1C4-ELISA, with (data not shown) or without the activating murine monoclonal anti–ADAMTS-13 antibody 17G2, if ADAMTS-13 antigen concentration was >0.030 μg/mL. A conformation index of <0.5 corresponds to a closed ADAMTS-13 (black-filled circles), while a conformation index of ≥0.5 corresponds to an open ADAMTS-13 (open circles). The dotted red line (conformation index, 0.5) indicates the cutoff value between closed and open ADAMTS-13. The blue arrows indicate pre-emptive immunosuppressive treatment during a biological relapse of ADAMTS-13. The green arrows indicate immunosuppressive treatment as part of the management of a clinical relapse of iTTP. Ag, antigen; b-RL, biological relapse; c-RL, clinical relapse; CI, conformation index; CsA, cyclosporin A; iTTP, immune-mediated thrombotic thrombocytopenic purpura; RTX, rituximab.

Fifteen patients with iTTP (8 women and 7 men) were included in the study, with a median follow-up of 7 years (IQR, 6-12 years), corresponding to 88 months (IQR, 74-143 months). During the follow-up period from 2008 to 2020, a total of 15 clinical relapses and 39 ADAMTS-13 relapses were observed, resulting in a median annual relapse rate of 0.50 (IQR, 0.23-0.62) (Table 1). Patients were stratified into 2 groups: group 1 (patients with a low relapse rate; annual relapse rate, <0.50, *n* = 8) and group 2 (patients with a higher relapse rate; annual relapse rate, ≥0.50, *n* = 7), with corresponding median relapse rates of 0.23 (IQR, 0.13-0.41) and 0.64 (IQR, 0.59-0.67; *P* = .0003) (Table 1; Figure 1A). Annual ADAMTS-13 activity, defined as the mean value per year, was not significantly different between groups (54.1 [IQR, 51.7-83.2] vs 50.0 [IQR, 38.8-57.7] IU/dL; *P* = .1893). All patients achieved at least once clinical remission, that is, ADAMTS-13 activity recovery. Interestingly, ADAMTS-13 activity recovery between 2 relapses (either clinical relapses or ADAMTS-13 relapses) occurred more consistently in group 1 (16/17 relapses, 94.1%) than that in group 2 (26/37 relapses, 70.3%; *P* = .0503), indicating a more durable ADAMTS-13 remission in group 1 (Table 2; Figure 2).

**Table 1:** Severity profile of 15 patients with iTTP included in the kinetic study during their follow-up.

Severity profile	Group 1	Group 2	Total	*P*
No. of patients	8	7	15	NS
Time of kinetic study (y)	9 (7-11)	7 (6-11)	7 (6-11)	.7624
Time of kinetic study (mo)	106 (80-143)	87 (73-141)	88 (74-143)	.7624
No. of all relapses	17 (0-5)	37 (3-8)	54 (0-8)	.0085^a^
No. of biological relapses	9 (0-3)	30 (3-7)	39 (0-7)	.0008^a^
No. of clinical relapses	8 (0-2)	7 (0-3)	15 (0-3)	.9907
Relapse rate per year	0.23 (0.13;0.41)	0.64 (0.59-0.67)	0.50 (0.23-0.62)	.0003^a^
Splenectomy	0	2 (1 before enrollment)	2	.4615
Pre-emptive therapies				
Rituximab	7	7	14	NS
Intensive rituximab	1	3	4	.5594
Cyclosporine A	0	3	3	.1923
Ofatumumab	0	1	1	.4615
Other	0	1	1	.4615

Data are presented as median (IQR) or total number (minimum-maximum per patient). Statistical analysis: Mann–Whitney U-test.

NS, nonsignificant.

a Statistically significant.

**Table 2:** Severity profile of 15 patients with iTTP included in the kinetic study during their follow-up.

Severity profile	Group 1	Group 2	Total	*P*
No. of patients with at least 1 occurrence of ADAMTS-13 activity normalization across TTP episodes	8/8 (100.0)	7/7 (100.0)	15/15 (100.0)	NS
No. of samples with a closed ADAMTS-13 conformation among samples collected prior to relapse in which ADAMTS-13 activity is ≥50 IU/dL	9/16 (56.3)	9/26 (34.6)	18/42 (42.3)	.1689
No. of samples with an open ADAMTS-13 conformation among samples collected prior to relapse in which ADAMTS-13 activity is ≥50 IU/dL	7/16 (43.8)	17/26 (65.4)	24/42 (57.1)	.1689
Time interval between ADAMTS-13 activity of ≥50 IU/dL with open ADAMTS-13 conformation and relapse (mo)	21 (17.5-48.5)	5 (2.0-11.0)	8.5 (3.5-15.8)	.0005^a^
ADAMTS-13 activity per year	54.1 (51.7-83.2)	50.0 (38.8-57.7)	52.5 (45.9-68.5)	.1893
Proportion of time spent in an open conformation	0.2 (0.1-0.5)	0.6 (0.4-0.7)	0.4 (0.1-0.7)	.1427

Data are presented as median (IQR) or *n* (%). Statistical analysis: Mann–Whitney U-test or chi-squared test.

NS, nonsignificant.

a Statistically significant.

### 3.2 Longitudinal follow-up and ADAMTS-13 conformation

Among the 479 samples available, 471 (98.3%) had an ADAMTS-13 antigen concentration >0.03 μg/mL, allowing ADAMTS-13 conformation analysis. An open conformation was observed in 98.1% of samples with ADAMTS-13 activity <30 IU/dL (107/109 samples) and in 42.0% (152/362) of samples with ADAMTS-13 activity >30 IU/dL (Figure 1B). At the time of ADAMTS-13 relapse or clinical relapse, ADAMTS-13 conformation was open in 46 of 47 (97.8%) tested samples. Among 39 complete or partial remission samples (no iTTP symptoms and ADAMTS-13 activity of ≥50% or ≥20 IU/dL, respectively) collected within 6 months prior to relapse, ADAMTS-13 conformation was open in 30 (77%) samples. In group 1, ADAMTS-13 conformation was open in 8 of 12 samples (66.6%), while in group 2, it was open in 22 of 27 samples (81.5%; *P* = .4161, nonsignificant) (Figure 2). Although not statistically significant, the higher proportion of open ADAMTS-13 conformation in group 2, where biological remission between episodes was less frequently observed, may suggest a possible trend toward an association between a more persistent open conformation during remission and a higher risk of relapse, which warrants further investigation in larger cohorts.

### 3.3 Association between normal ADAMTS-13 activity (≥50 IU/dL) and ADAMTS-13 conformation

Further, we investigated the risk of relapse in patients with complete ADAMTS-13 remission (ie, activity of ≥50 IU/dL), by comparing ADAMTS-13 conformation from samples of patients of both groups. An open ADAMTS-13 conformation was reported in 44 of 140 (31.4%) samples in patients of group 1 and in 35 of 106 (33.0%) samples in patients of group 2 (*P* = .8).

Focusing on samples collected within 6 months before relapse in patients with complete ADAMTS-13 remission, a closed conformation was observed more frequently in samples of patients from group 1 (9/16, 56.3%) than that in samples of patients from group 2 (9/26, 34.6%; *P* = .17), suggesting a possible trend in which a persistent open ADAMTS-13 conformation during remission may be associated with an increased risk of relapse. Furthermore, the median time to relapse in patients with complete ADAMTS-13 remission and an open ADAMTS-13 conformation was notably shorter for patients of group 2 than for patients of group 1 (5 months [IQR, 2.0-11.0 months] vs 21 months [IQR, 17.5-48.5 months], respectively; *P* = .0005), indicating that in group 2, relapse might occur more quickly after detection of an open conformation despite normalized activity, possibly reflecting a less stable remission. There was also a nonsignificant trend for a longer time with an open ADAMTS-13 conformation in group 2 than that in group 1 (0.6 [IQR, 0.4-0.7] vs 0.2 [IQR, 0.1-0.5], respectively; *P* = .1427). Finally, 1 patient in group 1 (case KIN-18) showed a consistently closed ADAMTS-13 conformation during remission and remained relapse free throughout follow-up, supporting that a stable closed conformation may be linked to sustained remission (Figure 2A).

### 3.4 Immunomodulatory therapies

During ADAMTS-13 relapses, anti-CD20 therapy has been shown to be an effective long-term treatment [24]. Rituximab was used as the first-line immunomodulatory therapy in all patients. Figure 2 illustrates the long-term kinetics of ADAMTS-13 activity and conformation, with treatments, over several years. Pre-emptive rituximab treatment upon a biological relapse of ADAMTS-13 allowed for the normalization of ADAMTS-13 activity within 1 month following the injection, leading to the sustained protection over several years, for patients of group 1 (Table 1; Figure 2A). However, in group 2, patients more frequently required alternative or intensified therapies for those with relapsing or refractory disease, defined as an insufficient clinical or biological response despite standard treatment (Table 1; Figure 2B). Three patients received an intensive rituximab regimen consisting of repeated infusion of rituximab (annually or every 3-6 months) to achieve biological remission of ADAMTS-13. Three patients received the calcineurin inhibitor cyclosporine A as an immunosuppressive agent in remission to normalize ADAMTS-13 activity. One of them also received ofatumumab, an anti-CD20 IgG1κ humanized monoclonal antibody, shortly after his latest biological relapse. Two refractory patients with iTTP underwent splenectomy: 1 before enrollment in the study, 4 years after their first episode of iTTP; and 1 during the study, 2 years after their first episode of iTTP. However, splenectomy did not result in sustained normalization of ADAMTS-13 activity and required further immunosuppressive therapy.

## 4 Discussion

This study reports distinct relapse phenotypes in iTTP, distinguishing patients with stable remission from those with frequent relapses. While longitudinal ADAMTS-13 activity measurement provides valuable insights into disease control, this parameter alone did not discriminate between patients with different relapse risks. By contrast, monitoring ADAMTS-13 conformation during long-term follow-up revealed more informative profiles, particularly in patients with higher relapse rates.

Patients classified as high relapsers (group 2) experienced more frequent relapses, shorter intervals between normalization and recurrence, and longer cumulative time spent with an open ADAMTS-13 conformation, even when activity was within normal range. These observations suggest that persistent or recurrent open conformation may reflect ongoing subclinical immunologic activity, potentially mediated by low levels of anti–ADAMTS-13 antibodies that are not detectable by ELISA assays. This aligns with prior evidence that an open conformation of ADAMTS-13 can precede the drop in its activity and may serve as an early biological marker for relapse.

Our work is in agreement with prior studies linking an open conformation of ADAMTS-13 to earlier relapses and emphasizes its potential as a predictive biomarker of relapse in patients with iTTP [29]. Our results align with those from the study by Prasannan et al. [30], who demonstrated the predictive value of ADAMTS-13 conformation in iTTP relapse management. In their study, patients with a closed ADAMTS-13 conformation at peak activity (ie, activity of >60 IU/dL) had significantly lower relapse rates than those with an open conformation within 1 year (9% vs 46%, respectively) and 2 years (23% vs 62%, respectively) [30]. Our results suggested a potential association between open conformation and shorter relapse intervals, particularly in patients with a high relapse tendency (group 2). Both studies highlight the role of ADAMTS-13 conformation as a biomarker of disease activity and relapse risk. However, while Prasannan et al. [30] highlighted peak ADAMTS-13 activity as a critical time point for ADAMTS-13 conformation test, we focused on the longitudinal dynamics of conformation changes throughout remission. This distinction may provide complementary insights into the prediction of the risk of relapse and the timing of pre-emptive treatments, such as anti-CD20 therapies [20,[23], [24], [25]]. The association between open conformation and relapse is also supported by De Waele et al. [29], who found similar trends in a large cohort of patients with iTTP. Our data reinforce these conclusions while adding the nuance that the duration and timing of open conformation during remission, rather than a single measurement, may be critical to stratify relapse risk. The hypothesis that an open conformation reflects the presence of ADAMTS-13 with autoantibody immune complexes, even in the absence of detectable free antibodies, needs further investigation. Since routine ELISA assays detect only free anti–ADAMTS-13 IgG, the conformation assay may provide indirect evidence of immune activity. In this context, conformation testing could be a biological marker of early immunologic relapse.

Our study has several strengths: a well-characterized cohort of patients with iTTP, with a median follow-up period of 9 years, with standardized monitoring and approximately 500 plasma samples analyzed. Although retrospective, the monitoring protocol was preestablished as part of routine care, ensuring the reliability of the data collected. The monocentric design of the study allowed for consistent clinical and laboratory assessments, providing data quality. Although the small number of patients included is limited, reducing statistical power of subgroup analyses and nonsignificant trends, the large number of plasma samples analyzed provides valuable longitudinal data. This study is as an important first step and a cornerstone for future larger-scale investigations.

Our study suggests that ADAMTS-13 conformation monitoring may provide complementary information to ADAMTS-13 activity measurements in predicting relapse risk in iTTP. A persistent or recurrent open conformation, even when ADAMTS-13 activity is normal, could potentially identify patients at higher risk of relapse. These preliminary findings suggest that ADAMTS-13 conformation may guide follow-up strategies and optimal timing of interventions. Future prospective studies with larger cohorts are needed to validate these observations and to better understand the immunologic mechanisms linking ADAMTS-13 conformation state to relapse. While ADAMTS-13 conformation testing has the potential to support more individualized monitoring and management strategies in iTTP, its clinical utility must be carefully assessed. Specifically, the performance of ADAMTS-13 conformation testing (including sensitivity, specificity, positive predictive value, and negative predictive value) will need to be evaluated in larger multicentric studies before it can be considered for clinical application.
